# The identification and molecular mechanism of anti-stroke traditional Chinese medicinal compounds

**DOI:** 10.1038/srep41406

**Published:** 2017-01-24

**Authors:** Jia-Qian Liu, Shao-Xing Dai, Jun-Juan Zheng, Yi-Cheng Guo, Wen-Xing Li, Gong-Hua Li, Jing-Fei Huang

**Affiliations:** 1 State Key Laboratory of Genetic Resources and Evolution, Kunming Institute of Zoology, Chinese Academy of Sciences, Kunming, 650223, China; 2 Kunming College of Life Science, University of Chinese Academy of Sciences, Kunming, 650204, China; 3 Institute of Health Sciences, Anhui University, Hefei, 230601, China; 4 Chinese University of Hong Kong Joint Research Center for Bio-resources and Human Disease Mechanisms, Kunming, 650223, China; 5 KIZ-SU Joint Laboratory of Animal Models and Drug Development, College of Pharmaceutical Sciences, Soochow University, Kunming, 650223, China; 6 Kunming Biological Diversity Regional Center of Instruments, Kunming Institute of Zoology, Chinese Academy of Sciences, Kunming, 650223, China

## Abstract

Stroke is a worldwide epidemic disease with high morbidity and mortality. The continuously exploration of anti-stroke medicines and molecular mechanism has a long way to go. In this study, in order to screen candidate anti-stroke compounds, more than 60000 compounds from traditional Chinese medicine (TCM) database were computationally analyzed then docked to the 15 known anti-stroke targets. 192 anti-stroke plants for clinical therapy and 51 current anti-stroke drugs were used to validate docking results. Totally 2355 candidate anti-stroke compounds were obtained. Among these compounds, 19 compounds are structurally identical with 16 existing drugs in which part of them have been used for anti-stroke treatment. Furthermore, these candidate compounds were significantly enriched in anti-stroke plants. Based on the above results, the compound-target-plant network was constructed. The network reveals the potential molecular mechanism of anti-stroke for these compounds. Most of candidate compounds and anti-stroke plants are tended to interact with target NOS3, PSD-95 and PDE5A. Finally, using ADMET filter, we identified 35 anti-stroke compounds with favorable properties. The 35 candidate anti-stroke compounds offer an opportunity to develop new anti-stroke drugs and will improve the research on molecular mechanism of anti-stroke.

Stroke, cerebral infarction, has been defined as a rapid onset clinical syndrome of the central nervous system deficit due to ischemia based on neuroimaging, neuropathological and clinical evidence of permanent cell injury^1^,^2^. Stroke can be generally divided to two types, ischemic and hemorrhage stroke. Ischemic stroke accounts for approximately 80% of strokes, and it often occurs in the middle cerebral artery^3^. When the cerebral artery in or leading to brain ruptures is clogged by thrombus, atherosclerotic plaque or other particle, nerve cells in this brain region will die in a few minutes due to the deficiency of oxygen supply^4^. Without therapeutic intervention in time, the ischemic region will undergo irreversible damage and dysfunction. Brain injury area continues to spread out and get broader.

A number of common and significant molecular and cellular responses related to post-ischemia have been mentioned, such as up-regulated gene expression^5^ (c-*fos*, c-*jun*, AP-1, NF-κB) and apoptosis^6^. Therefore, interruption of these processes by antagonizing some potential protein targets may contribute to the improvement of novel stroke therapies. Current therapeutic targets for stroke treatment play variety of biological function in stroke process, such as, acetylcholinesterase (AChE), angiotensin I converting enzyme 2 (ACE2), P2Y purinoceptor 12 (P2Y12), postsynaptic density protein 95 (PSD-95), peroxisome proliferator-activated receptor gamma (PPAR-γ), plasminogen activator inhibitor-1 (PAI-1). AChE binds on the post-synaptic membrane and terminates neurotransmission^7^,^8^. ACE2 is predominantly expressed in heart and kidney vascular and has direct effective cardiac function^9^. P2Y12 is mainly expressed on blood platelets membrane and acts on blood clotting^10^. PSD-95 is exclusively located in the post-synaptic density of neurons and plays a major role in synaptic plasticity^11^,^12^. PPAR-γ works as a transcription factor and regulates genes expression^13^,^14^. PAI-1 inhibits fibrinolysis and functions as a risk factor for thrombosis and atherosclerosis^15^,^16^.

As a high morbidity and mortality cerebrovascular disease, stroke has various risk factors. The two major risk factors are atherothrombosis and arterial hypertension. In addition, diabetes mellitus, cardiac diseases, smoking, alcohol intake, obesity and psychosocial stress and depression all these risk factors contribute to stroke^17^. Accordingly, risk reduction becomes the most significant preventing method for first-onset or recurrent stroke.

The treatment of stroke should be initiated within three hours of symptoms to sudden appearance, in order to prevent irreversible injury of neuronal cells^18^. Although there is significant progress in the treatment of stroke in recent years, it is undeniably that prevention is still the best approach to reduce stroke morbidity and mortality^19^. Known drug types for the treatment and prevention of stroke include thrombolytics, anticoagulants, antiplatelet drugs, neuroprotective agents, calcium channel blockers and free radical scavengers^20^,^21^. Although different kinds of drugs are marketed for anti-stroke, but these drugs often have side effects and the mechanism of interaction is not clear. Therefore, it is significant to find some efficient compounds with low toxicity for stroke treatment.

In TCM (Traditional Chinese Medicine) Database@Taiwan, there are more than 60000 compounds isolated from thousands of TCM ingredients^22^. Both CDX (2D) and Tripos Mol2 (3D) formats of each pure compound in the database are available for download and virtual screening^22^. The large number of natural compounds provides a favorable basis for the screening of anti-stroke drugs.

In this study, we screened the TCM database to find available compounds that can actively interact with the anti-stroke target using molecular docking. As the work flow (Fig. 1), we obtained compounds for all anti-stroke targets through molecular docking and get candidate anti-stroke compounds together with three validation methods. After ADMET filter, some of candidate compounds have favorable properties as drugs. Our research aims to identify the plant-derived compounds which have the anti-stroke activity. At the same time, through network pharmacology method to explore the molecular mechanism of traditional Chinese medicine for the treatment of stroke.

## Results

### Docking results of the target with compounds

In 60000 TCM compounds, totally 30438 compounds which have plant information were remained for molecular docking. Docking results of the compounds and marketed anti-stroke drugs to 15 targets were show in Table 1. Many TCM compounds and Intergrity drugs were successfully docked to the 15 anti-stroke targets. The docking energy of ligand which embedded in the crystal structure ranged from −13.6 to −4.4 (kcal/mol). The variation of lowest energy for the 15 targets ranged from −16.2 to −8.5 (kcal/mol).

For target SOD1, totally 21940 compounds are predicted to bind it. Its lowest docking energy and embedded ligand docking energy are −8.5 and −4.4 (kcal/mol), respectively. The target PSD-95 doesn’t have an appropriate embedded ligand in crystal structure, so that there is no embedded ligand docking energy as a reference value. But the lowest energy of this target (−11.8 kcal/mol) is comparable to that of other targets.

### The enrichment of compounds in anti-stroke Chinese medicine plants

As there are so many TCM compounds binding to anti-stroke targets, we just took top 1% compounds for each target as the candidate anti-stroke compounds.

In order to confirm these candidate anti-stroke compounds, we analyzed the enrichment of these candidate compounds in anti-stroke plants which have widely clinical usage in China. Nearly 1000 anti-stroke Chinese medicine prescription were collected by manually checking more than 5000 articles about clinic study of stroke treatment (Supplementary Table S1 and Supplementary Dataset 1). After the statistical analysis of these prescriptions, 192 anti-stroke plants were identified (Supplementary Table S2). The collected 192 anti-stroke plants belong to 18 categories according to the functional property in the TCM database. Most of these plants are included in three categories, ‘tonifying and replenishing’, ‘heat-clearing’, ‘blood-activating and stasis-resolving’ (Supplementary Fig. S1).

**Figure 2:**
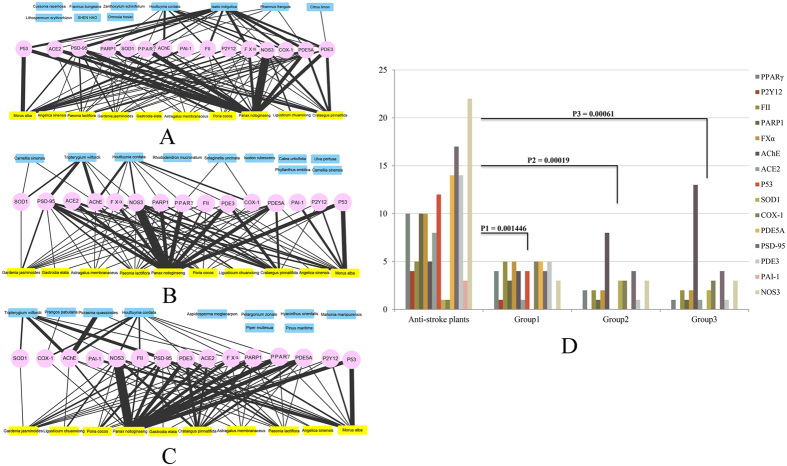
The top 1% compounds are significantly enriched in anti-stroke plants. **(A~C)** The interaction network of anti-stroke targets with anti-stroke plants and Group1, Group2, Group3 plants. The line’s thickness indicates the number of compounds, and the number ranges from 1 to 12. Pink, yellow and blue colors, respectively represent anti-stroke target, anti-stroke plants and random non-anti-stroke plants. (**D)** The significant enrichment difference of top 1% compounds for each target between anti-stroke plants and non-anti-stroke plants (the T-test P-value < 0.01 means significantly difference).

Next, the top 10 anti-stroke plants and 3 groups of 10 random selected non-anti-stroke plants were used to make enrichment analysis of the top 1% compounds for each target. Three networks had been constructed (Fig. 2A~C). In these networks, it is easy to find that top 1% compounds for each target are mostly enriched in anti-stroke plants, with only a small part in non-anti-stroke plants. The enrichment of top 1% compounds for each target between two kinds of plants has significant difference. The P-values are 0.001446, 0.00019 and 0.00061 for the comparisons between anti-stroke plants and Group1 plants, Group2 plants, Group3 plants, respectively (Fig. 2D).

### The similarity between candidate anti-stroke compounds and existing drugs

After calculation of similarity between top 1% compounds and existing drugs, we find some candidate anti-stroke compounds are structurally identical with existing drugs (Tc = 1). A network was constructed to demonstrate the connection among targets, candidate anti-stroke compounds and existing drugs.

**Figure 3:**
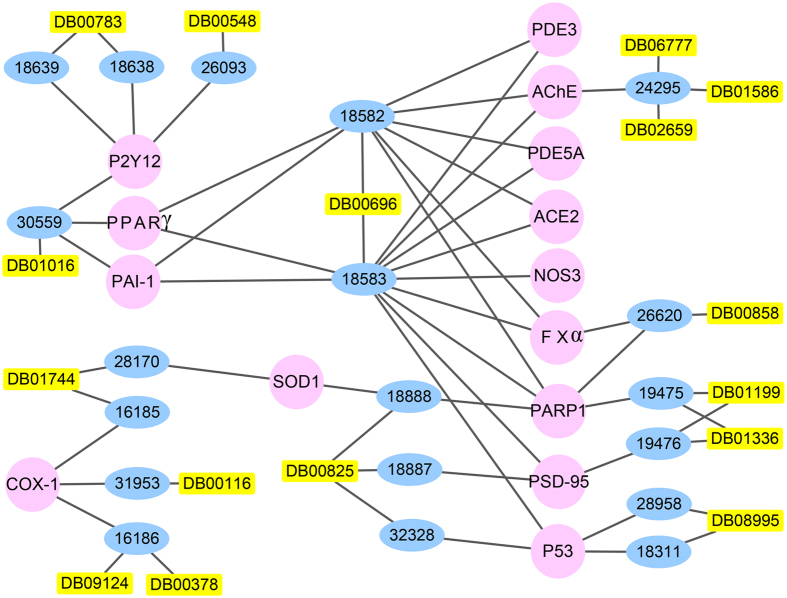
The candidate anti-stroke compounds and existing structurally identical drugs. The network contained anti-stroke targets, TCM compounds and structurally identical drugs. Some compounds and drugs can act on one target, and others can act on more than 3 targets. The detail information of the three can be found in Supplementary Dataset 1.

In the network, there are 16 drugs, 19 compounds and 14 anti-stroke targets (Fig. 3). Compounds 18639-18638-26093, 28958-18311, 31953-16186 and 24295 can only interact with one target P2Y12, P53, COX-1 and AChE, respectively. Their corresponding drugs are DB00783 (estradiol), DB00548 (azelaic acid); DB08995 (diosmin); DB09124 (medrogestone), DB00378 (dydrogesterone) and DB00116 (tetrahydrofolic acid); DB06777 (chenodeoxycholic acid), DB02659 (cholic acid) and DB01586 (ursodeoxycholic acid), respectively (Supplementary Table S3). On the contrary, compounds 18582 (ergotamine) and 18583 (ergotaminine) have 10 common targets (PDE3, AChE, PDE5A, ACE2, FXα, PARP1, PSD-95, P53, PPARγ and PAI-1) and share the same structurally identical drug ergotamine (DB00696) which also relate with target NOS3 through compound 18583 (ergotaminine). DB01016 (glyburide) can relate with target P2Y12, PPARγ and PAI-1 through compound 30559 (genameside D). DB00825 (menthol) can relate with target PARP1, PSD-95 and P53 through three compounds respectively. Another four drugs all can relate with two targets through their structurally identical compounds.

### The target cluster of candidate anti-stroke compounds

With further data analysis, the top 1% compounds actually have 2355 candidate anti-stroke compounds after removing duplication. In terms of interacting with anti-stroke targets, these 2355 compounds have common characteristics. There are 1564 compounds with single target and 791 compounds with multiple targets in total.

**Figure 4:**
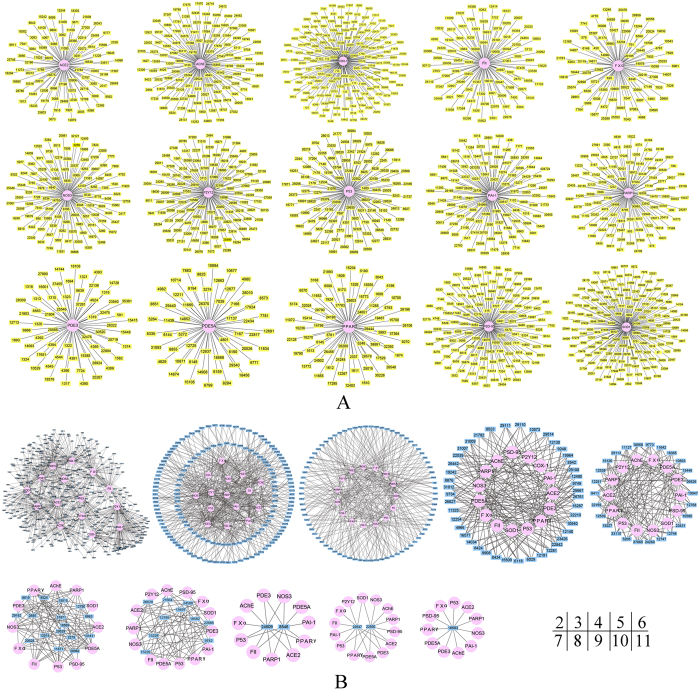
Candidate anti-stroke compounds act on their target in a characteristic way. **(A**) The single target candidate anti-stroke compounds with single target. The radial circle diagram shows single target compounds colored by yellow and their only anti-stroke target colored by pink. (**B**) Nine networks of anti-stroke compounds with multiple targets. The compounds in each network have the same number of targets. In the bottom right corner, the number means the targets number of compounds in each network. Compounds are displayed by small blue rectangles.

Different anti-stroke targets have different numbers of single-target compounds (Fig. 4A). Target COX-1 has the largest number of compounds 172, target SOD1 has 158 compounds, target PSD-95 has 136 compounds and target AChE has 123 compounds. These single target compounds are colored by yellow.

Besides, there also have multiple targets compounds. Candidate anti-stroke compounds and their targets were assigned to different clusters depend on the different target number of each compound (Fig. 4B). In two-target cluster, it has 456 compounds. Three-target and four-target clusters have 310, 78 compounds, respectively. Only one compound 18583 can act on 11 anti-stroke targets.

### The structure cluster of candidate anti-stroke compounds

**Figure 5:**
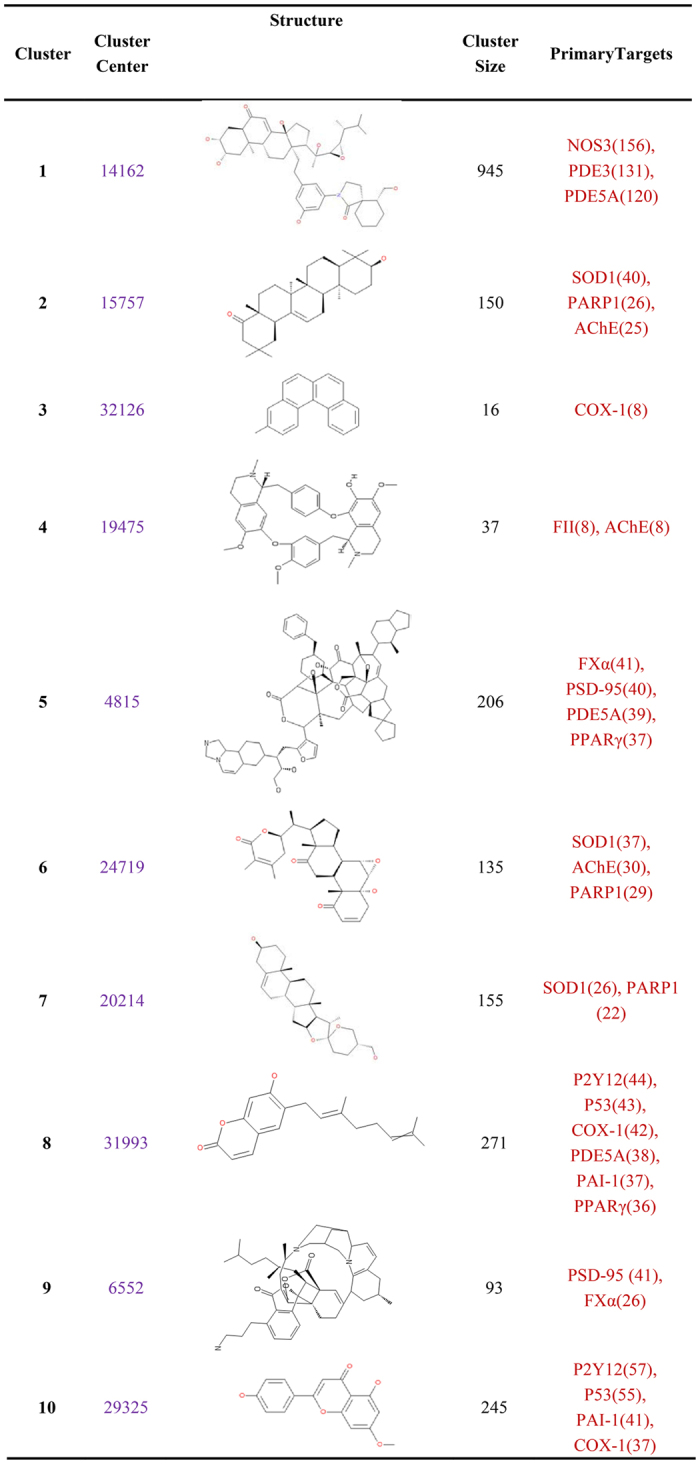
The 10 anti-stroke TCM compounds clusters and their primary targets. There are 10 clusters in this figure. Cluster membership property is given as cluster center. The compound ID and representative structure of the cluster center for each cluster are shown. Cluster size means the number of membership in each cluster. Number behinds target means the number of compounds interacted with this target.

In order to find out the relationship among the candidate anti-stroke compound, cluster ligands protocol in Pipeline Pilot was used to recognize the common pattern of 2355 candidate anti-stroke compounds. After clustering, all compounds are assigned into 10 clusters (Fig. 5). Cluster center compounds all contain the carbocyclic structure. In order to show the relationship between the cluster center compounds and the other cluster members, an edge-weighted force directed layout method have been used in the network (Supplementary Fig. S2). The distance (or similarity) between the cluster center compounds and the other cluster members are represented as edge thickness. The similarity is proportional to the edge thickness. Each cluster has different number of compounds, the smallest cluster has only 16 compounds, while the largest cluster contains up to 947 compounds. Cluster3 and cluster9 both have nearly half of compounds (50%, 44.1%) acting on their primary target, even they almost have the littlest cluster size (16, 93). The other clusters have various cluster size (from 37 to 945) but the number of its members acting on their primary target dose not changed widely (16.2~26.7). Cluster2, cluster6 and cluster7 share the common primary target SOD1. Cluster5 and cluster9 also have the common primary target PSD-95. Target P2Y12, P53 are the common primary target of cluster8 and cluster10.

### The candidate anti-stroke compounds and their molecular mechanism

**Figure 6:**
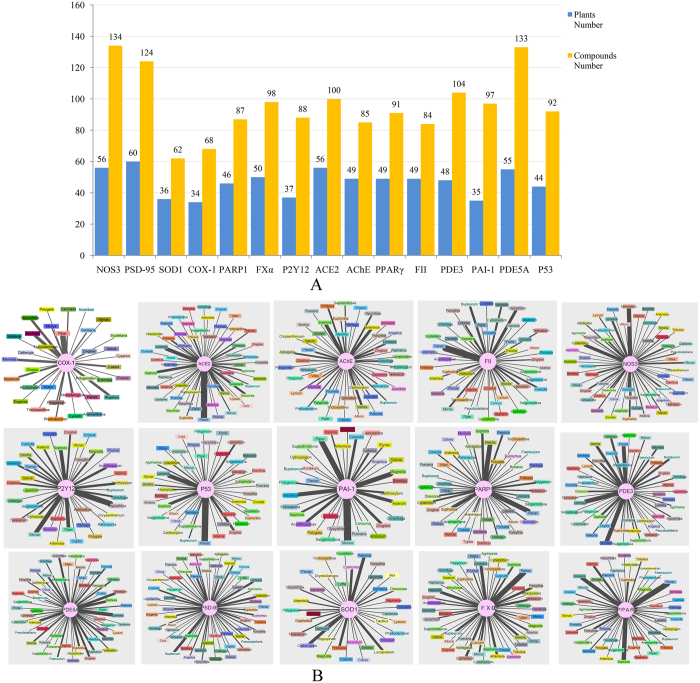
Numbers of anti-stroke plants and compounds for each anti-stroke target and network of anti-stroke targets and plants. **(A**) The exact number of anti-stroke plants and compounds for anti-stroke targets. The number is tagged above each column, and every target is displayed on the horizontal axis. (**B**) Network of anti-stroke targets and plants. Anti-stroke targets actually connect with many different anti-stroke plants. Small rectangles of different colors represent each plant marked their name. Target ranges from left to right according to their candidate anti-stroke compounds number. The line’s thickness indicates the number of compounds, and the number ranges from 1 to 12.

In order to explore the molecular mechanism of candidate anti-stroke compounds, 2355 candidate anti-stroke compounds were mapped to the 192 anti-stroke plants (Fig. 6). Consequently, the result shows that the plant number for each target ranges from 34 to 60 (Fig. 6A). However, in these plants, the number of candidate anti-stroke compounds for each targets dramatically reaches a maximum of 134 (target NOS3) and a minimum of 62 (target SOD1) (Fig. 6A). Target PSD-95 gets the larger compounds number 124 and the largest plants number 60. Targets PDE5A also obtain larger number of candidate anti-stroke compounds 133. Target SOD1 and COX1 not only have the smallest plants number (36, 34), but also have the smallest compounds number (62, 68). The number of anti-stroke plants is not always consistent with the number of candidate anti-stroke compounds, such as target AChE and FII (Fig. 6B). For target ACE2, FII, P53, FXα and PPARγ, the anti-stroke plant *Panax* contains largest number of compounds (12, 11 and 10, respectively) interacted with these targets. The anti-stroke plant *Morus* and *Piper* contain 11 and 9 compounds which can interact with target PAI-1 and COX1, respectively.

### The 35 candidate anti-stroke compounds with favorable ADMET characteristics

After ADMET filtering, there are 35 candidate anti-stroke compounds remained from 2355 candidate anti-stroke compounds (Supplementary Table S4). Among these compounds, compounds 23116 can interact with three targets ACE2, FXα and PARP-1; compounds 8619 (dianthins d), 9908 (tonkinochromane c), 26629 (blestrin D), 28468 (dibothrioclinin II) can interact with three or more targets. Compound 23116 (rockogenin) and 26624 (blestriarene A) have common target ACE2 and PARP1. Compound 8625 ((S)-suspensaside methyl ether), 33377 (kaempferol-3-O-β-D-glucopyranosyl (1 → 2)-β-D-6-acetylglucopyranoside) and 9438 (shancilin) are have three targets.

The other 26 compounds are all single-target compounds, 6 of them interact with target SOD1. 4 of them interact with target PARP-1, 4 of them interact with target FXα, 3 of them interact with target FII, COX-1 and 2 of them interact with target ACE2, P2Y12. Target AChE and PAI-1 also get one single-target compound. Most of these 26 compounds still have not been used for anti-stroke research.

## Discussion

Along with TCM compounds, Integrity anti-stroke drugs were also docked to targets. The docking energy of drugs is almost higher than the docking energy of embedded ligand and top 1% compounds (Supplementary Fig. S3A). In network, Integrity drugs have different targets, such as PPARγ modulators, FXα inhibitors, K^+^ (ATP) channel blockers, signal transduction modulators, lipid peroxidation inhibitors and antiplatelet (Supplementary Fig. S3B). These drugs were well docked to their known anti-stroke target. It indicates that the docking results are reliable and the top 1% docked compounds can be taken as candidate anti-stroke compounds.

Therefore, the 2355 compounds, obtained from the top 1% compounds of each target, can take as candidate anti-stroke active compounds for each target. These 2355 compounds can be clustered to different categories, through their interacted anti-stroke targets (Fig. 4) and their structure descriptors (Fig. 5). The discovery of the association between compounds and targets can easy satisfy various anti-stroke drugs design for different purpose. For example, the cocktail drug that acts to more than one target and has multiple effects.

The identical structure between existing drugs in DrugBank database and the 2355 candidate anti-stroke compounds can give huge enlightenment to drug exploration of stroke treatment. On the one hand, existing drugs have already been applied to anti-stroke, like estradiol (DB00783)^23^, progesterone (DB00378, DB09124)^24^,^25^, ergotamine (DB00696)^26^ and glyburide (DB01016)^27^. Candidate anti-stroke compounds that have identical structure with these drugs may also have anti-stroke function through interacting with their targets (Fig. 3). Menthol (DB00825) and azelaic acid (DB00548) are likely to have new function that useful to anti-stroke. It also suggests that we find the right anti-stroke research object, these 2355 candidate compounds.

According to the enrichment result, target NOS3, PSD-95, PDE3, PDE5A and P53 have large number of compounds in anti-stroke plants (Fig. 2D). We noted that some non-anti-stroke plants in Groups 2 and 3 have more compounds can interact on target AChE. It may be the truth as some non-anti-stroke plants may became anti-stroke plants with further research. In all 192 anti-stroke plants, there are also many compounds which can interact with target NOS3, PDE5A, PSD-95 (Fig. 6A). It shows that significant targets can interact with more anti-stroke compounds and plants. And the plant has more compounds which can interact with these targets; this means that the plant and their compounds are truly important for the anti-stroke target (Fig. 6B). These may unveil the anti-stroke mechanism of candidate anti-stroke compounds. Target NOS3 produces nitric oxide (NO) which is implicated in vascular smooth muscle relaxation through a cGMP-mediated signal transduction pathway and mediates vascular endothelial growth factor (VEGF)-induced angiogenesis in coronary vessels and promotes blood clotting through the activation of platelets^28^,^29^. Target PSD-95 is required for synaptic plasticity associated with NMDA receptor signaling^30^. Target PDE5A is a cGMP-specific phosphodiesterase which hydrolyzes cGMP to 5′-GMP and together with NOS3 involves in the regulation of intracellular concentrations of cyclic nucleotides and is important for smooth muscle relaxation in the cardiovascular system^31^. Besides, there are many candidate anti-stroke compounds interacted to these 3 targets (Fig. 4). Therefore, both the 3 anti-stroke targets and their candidate compounds are worthy to get more attention for the research of anti-stroke mechanism.

After five ADMET properties filtering, there is no Integrity drug left; but the 35 candidate anti-stroke compounds remained (Supplementary Table S4). Among the 35 compounds, there are 26 single-target compounds, 5 double-target compounds and 4 compounds with more than two targets. As their structures and targets have already known, these compounds can be directly applied to preclinical experiment of anti-stroke. Because of the favorable property (Supplementary Table S4), these 35 candidate anti-stroke compounds are expected to benefit in the research on anti-stroke. Whether the 35 compounds have a physiological anti-stroke function is the focus of future research on stroke treatment and prevention.

Usually, molecular docking results will be influenced by many factors and the accuracy of docking may change from 0% to 92.66%. Even a high docking score might be questionable^32^. During our research, we also consider the credibility of the molecular docking. So we used three validation methods to make the results convincible. Firstly, the clinical anti-stroke plants were used for validation. The candidate anti-stroke compounds were significant enriched the anti-stroke plants other than the random selected plants. Secondly, the embedded ligand in the crystal structure was set as reference. The docking energy of candidate anti-stroke compounds is better than or comparable with that of embedded ligand. Finally, some drugs structural identical with these candidate anti-stroke compounds have been reported in anti-stroke research. Therefore, our results are supported by both the validation methods and literatures. In the future, the molecular dynamics (MD) simulation will be applied to validate the key target-compound interaction and we will seek cooperation for experimental test of these candidate anti-stroke compounds.

## Methods

### Collection of TCM compounds, anti-stroke targets and existing drugs

We collected the information concerning the plants and plant-derived compounds from the TCM database. The relationship of the plant and its derived compounds was also collected. All compounds were downloaded as mol2 (3D) format. The format was converted to SMILES string by the Open Babel toolbox.

A total of 8529 plants (herb IDs) and more than 60000 compounds were collected and downloaded. Only 30438 compounds with herb IDs were identified. The Discovery Studio software was applied to filter the compound by ‘Lipinski and Veber Rules’. The targets for therapeutic intervention of stroke and drugs marketed for the prevention and treatment of stroke were gained from the Thomson Reuters Integrity database. According to Integrity stroke targets information, the structures of each target protein binding to its embedded ligands were obtained from PCSB Protein Data Bank. In order to compare the similarity of the candidate anti-stroke compounds with the existing drugs, 1985 approved drugs from DrugBank were downloaded.

### Prediction of the interaction between targets and compounds

AutoDock Vina v1.1.2 was used to predict the interaction of stroke targets and TCM compounds. The structures of these target proteins were prepared with AutoDock tools v1.5.6 as suggested in the user guide. The structural binding center of embedded ligand for each target was set as the docking center. To allow free rotation of the compounds, the search space was set to 25 × 25 × 25 Å in each axis. The format of input molecular should be pdbqt. All other docking parameters were set to the default values. Each docking is performed by a command that contains space size and three-dimensional coordinate of docking center. The binding pose with the lowest energy was selected as the best model for each docking test. The docking energy score decides whether the compound can effective interacts with targets, and targets have different docking energy level. Therefore top percentage compounds are more equal for the docking condition of all targets. So that we choose top 1% compounds of each target as the candidate anti-stroke compounds.

### Identification of anti-stroke plants

Three key terms “stroke”, “cerebral hemorrhage” and “cerebral infarction” were taken as ‘subject’ respectively to retrieve Chinese medicine prescription of anti-stroke from the related Chinese articles in CNKI database. Only articles of clinical study were remained. The articles of animal model study were ruled out. The Chinese medicine prescriptions and its usage frequency were collected from these articles. From the prescription, we can easily determine the anti-stroke plant. Chinese version of raw data prescription with correspond English includes key term, the Latin name of anti-stroke plants in each prescription, patient number (male and female if available), clinical research article title, published data (years) and reference format of articles. The information is contained in Supplementary Dataset 1 as a separate Excel file.

### Calculation of similarity between candidate anti-stroke compounds and existing drugs

The similarity between the candidate anti-stroke compounds and the existing drugs was calculated. The structural similarity was measured by Tanimoto coefficient (Tc). Tc is defined as Tc = C(i, j)/U(i, j), where C(i, j) is the number of common features in the fingerprints of molecules i and j, and where U(i, j) is the number of all features in the union of the fingerprints of molecules i and j. The fingerprint FP2 implemented in the Pybel were generated for each structure and used to calculate TC. Two compounds are considered structurally identical if their fingerprints have a Tc of 1.

### Clusters of candidate anti-stroke compounds

Clustering is the assignment of a set of molecules into subsets or clusters so that each molecule has similar properties in the same cluster. The cluster ligands protocol in BOVIA Pipeline Pilot V8.5 was used to cluster the 2355 candidate anti-stroke compounds. It is based on the root-mean-square (RMS) difference of descriptor properties or Tanimoto distance for fingerprints, or the combination of the two if both numeric descriptors and fingerprints are being used. Here, only the fingerprint FP2 was used. The clustering is done by a relocation method based on maximal dissimilarity partitioning. Cluster selection can be performed by size or by number. We assigned the cluster number as 10. The cluster center was selected if the sum of its distance to every member reaches a minimum value.

### Construction of two type networks

The software Cytoscape v3.2 was used to construct two types of network. The first type of network is about anti-stroke targets and plants. The plant and the target will be connected If the plant has compounds interacted with the target. The link strength is represented as the line’s thickness, which indicates the number of the compounds between the target and the plant. The random non-anti-stroke plants are chosen based on two conditions. Firstly, the selected plants are not in the list of 192 anti-stroke plants. The second condition is that the distribution of the number of compounds in the 10 selected non-anti-stroke plants is similar with that of the top 10 anti-stroke plants. The difference of the two distributions was tested with t-test. For this case, the p-value should more than 0.05, which indicates the two distributions have no significant difference. A python script was used to achieve the above process. The second type of network is about candidate anti-stroke compounds, their targets and structurally identical drugs. The drug and candidate anti-stroke compound will be connected if the compound with TC value of 1 to the drug. The compounds and their targets are also connected in this network. Beside, in Supplementary Fig. S2, the thickness of the line between nodes shows the molecular distance in each cluster.

### Estimation of ADMET properties for candidate anti-stroke compounds

The ADMET descriptors protocol in Discovery Studio software has been used to compute candidate anti-stroke compounds properties. We take five properties, aqueous solubility, blood brain barrier penetration (BBB), plasma protein binding (PPB), hepatotoxicity and human intestinal absorption (HIA) to filter TCM compounds. The favorable level had been controlled as follows. The parameter of aqueous solubility had been controlled on 2~4 (2: yes, low; 3: good; 4: optimal). The parameter of blood brain barrier penetration (BBB) had been controlled on 0~2 (0: very high penetrant; 1: high; 2: low). The parameter of hepatotoxicity had been controlled on FALSE. The parameter of plasma protein binding (PPB) controlled on FALSE. The parameter of human intestinal absorption (HIA) had been controlled on 0~1 (0: good; 1: moderate).

## Additional Information

**How to cite this article:** Liu, J.-Q. *et al*. The identification and molecular mechanism of anti-stroke traditional Chinese medicinal compounds. *Sci. Rep.* **7**, 41406; doi: 10.1038/srep41406 (2017).

**Publisher's note:** Springer Nature remains neutral with regard to jurisdictional claims in published maps and institutional affiliations.

## Supplementary Material

Supplementary Information

Supplementary Information

## Figures and Tables

**Figure 1:**
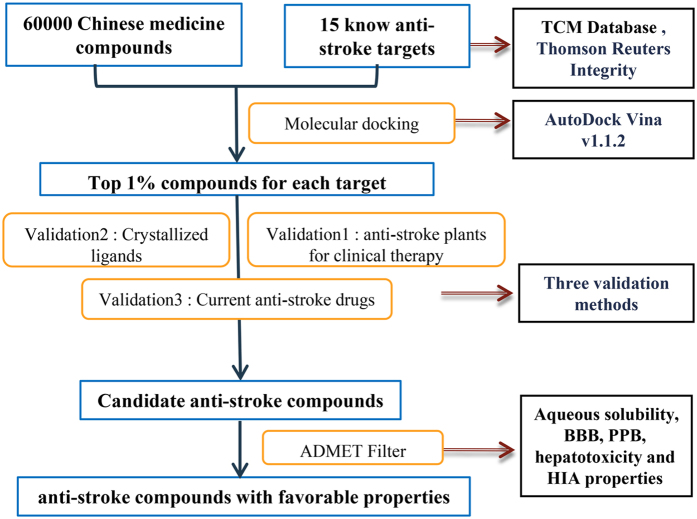
The work flow of this study. Compounds and anti-stroke target were obtained from TCM database and integrity, respectively. According to molecular docking energy, top 1% compounds for 15 targets were chosen. Clinical anti-stroke plants in Validation 1 support that compounds in these plants should have anti-stroke effect. Validation 2 makes sure the target structure and molecular docking pocket we taken are work, and docking energy of embedded ligand in the crystal structure can be taken as reference. Validation 3 shows that TCM compounds had better result in molecular docking. We do validation 2 and validation3 together with compounds docking. Using ADMET filter, we can get candidate anti-stroke compounds with favorable properties as drugs.

**Table 1:** 15 Anti-stroke targets with the number of successfully docked TCM compounds.

PCSB ID	Protein Name	Compound Number	Ligand Energy^a^	Lowest docking Energy	Reference
1Q4G	COX-1 (Cyclooxygenase-1, PTGS1)	125	−8.9	−9.1	33
1R4L	ACE2 (Angiotensin I converting enzyme 2)	18737	−7.3	−13.7	11,12
1SO2	PDE3 (Phosphodiesterase3)	616	−11	−14.7	34
3BJC	PDE5A (cGMP-specific phosphodiesterase 5A)	14084	−7.7	−14.1	35
3IA6	PPARγ (Peroxisome proliferator-activated receptors)	143	−9.8	−11.8	16,17
3WP0	PSD-95 (Postsynaptic density protein 95)	NA^a^	NA	−11.8	14,15
3ZME	P53 (Tumor protein P53)	1034	−8.3	−11.1	36
4 A7U	SOD1 (Superoxide dismutase [Cu-Zn])	21940	−4.4	−8.5	37,38
4AQH	PAI-1 (Plasminogen activator inhibitor-1)	2066	−7.7	−10.8	18,19
4BAQ	Coagulation factor II (Prothrombin)	458	−8.6	−11.3	39
4BTI	Coagulation factor X (Stuart factor)	1481	−9	−12.4	40
4D1P	NOS3 (Nitric oxide synthase 3)	4493	−9.7	−16.2	41
4M0F	AChE (Acetylcholinesterase)	18	−13.6	−14.7	9,10
4PY0	P2Y12 (P2Y purinoceptor 12)	4523	−7.9	−10.7	14
4ZZZ	PARP1 (Poly [ADP-ribose] polymerase 1)	17302	−8.2	−14.9	42

^a^This table includes the PCSB ID and protein name of anti-stroke targets. There also have references about the roles of these targets in anti-stroke treatment and prevention. ‘Ligand Energy’ means docking energy of ligand which embedded in the crystal structure. ‘NA’ means that the structure hasn’t available embedded ligand for docking. The average docking values (−8.7) of other embedded ligands had been taken as the value of target PSD-95.
