# Supplementary material for: The identification and molecular mechanism of anti-stroke traditional Chinese medicinal compounds
Source: Sci Rep. 2017 Jan 24;7:41406. doi: 10.1038/srep41406 (PMC5259785; doi:10.1038/srep41406)
Supplement: Supplementary Information [file srep41406-s1.pdf]

The identification and molecular mechanism of anti-stroke traditional Chinese medicinal compounds

Jia-Qian Liu[1,2,+], Shao-Xing Dai[1,2+], Jun-Juan Zheng[1,2,+], Yi-Cheng Guo[1], Wen-Xing Li[1,3], Gong-Hua Li[1,2*], and Jing-Fei Huang[1,2,4,5,6,*]

1 State Key Laboratory of Genetic Resources and Evolution, Kunming Institute of Zoology, Chinese Academy of Sciences, Kunming, 650223, China.

2 Kunming College of Life Science, University of Chinese Academy of Sciences, Kunming, 650204, China.

3 Institute of Health Sciences, Anhui University, Hefei, 230601, China.

4 Chinese University of Hong Kong Joint Research Center for Bio-resources and Human Disease Mechanisms, Kunming, 650223, China.

5 KIZ-SU Joint Laboratory of Animal Models and Drug Development, College of Pharmaceutical Sciences, Soochow University, Kunming, 650223, China.

6 Kunming Biological Diversity Regional Center of Instruments, Kunming Institute of Zoology, Chinese Academy of Sciences, Kunming, 650223, China

[+] These authors contributed equally to this work.

*Corresponding author:

Gong-Hua Li (ligonghua@mail.kiz.ac.cn)

Jing-Fei Huang (huangjf@mail.kiz.ac.cn)

Tel: +86 0871 65199200

Fax: +86 0871 65199200

Supplementary Information

Supplementary Table 1. Results of Chinese medicine clinical anti-stroke article retrieval. In this table, different research topics have different retrieval results. Cerebral infarction obviously has the largest number in every column. Retrieval article number means the total number retrived. Prescription article number means the whole number of the article in which conations Chinese medicine prescription and patient information.

Research Topic	Retrieval Article number	Prescription Article Number	Prescription Number	Patient Number
**Stroke Disease**	1302	83	134	6841
**Cerebral Infarction**	3078	570	618	35005
**Cerebral Hemorrhage**	1223	148	202	9012

Supplementary Table 2. The clinical anti-stroke plants categorized by properties. Anti-stroke plants belong to 18 properties. The Latin name of anti-stroke plants has been listed in the table.

1	**Exterior-Releasing**		Lycium		Agrimonia		Albizzia		Curculigo		Amomum
	Angelica		Stellaria	8	**Blood-A, Tasis-R**	11	**Orifice-Opening**		Cervus		Magnolia
	Vitex	3	**Purgative**		Ligusticum		DryobAlanops		Panax	17	**Interior-warming**
	Ligusticum		Rheum		Commiphora		Moschus		Polygonum		Cinnamomum
	Allium		Cannabis		Corydalis		Liquidambar		Rehmannia		Zanthoxylum
	Asarum		Prunus		Curcuma		Acorus		Angelica		Zingiber
	Chrysanthemum		Euphorbia		Boswellia	12	**Liver-P, Wind-E**		Paeonia		Evodia
	Mentha	4	**Dampness-R**		Leonurus		Tribulus		Polygonatum		Foeniculum
	Bupleurum		Alisma		Carthamus		Gastrodia		Lycium		Eugenia
	Pueraria		Polyporus		Spatholobus		Buthus		Ophiopogon	18	**No Category**
	Saposhnikovia		Poria		Salvia		uncaria		Asparagus		Pheretima
	Morus		Coix		Vaccaria		Centipede		Eclipta		Ostrea
	Notopterygium		Dianthus		Lycopus		Calculus		Dendrobium		Erigeron
	Cimicifugae		Plantago		Daemonorops		Bombyx		Ligustrum		Sinapis
2	**Heat-Clearing**	5	**Qi-Regulating**		Caesalpinia	13	**Tonifying, Replenishing**		Morus		Cristaria
	Prunella		Citrus		Drynaria		Codonopsis		Sesamum		Ilex
	Gardenia		Melia		Strychnos		Panax	14	**Astringent**		Styrax
	Cassia		Cyperus		Sparganium		Ziziphus		Ephedra		Akebia
	Trichosanthes		Lindera		Manis		Dioscorea		Schisandra		Luffa

	Anemarrhena		Aucklandia	9	**Cough-S, Panting-C**		Atractylodes		Terminalia		Oroxylum
	Phellodendron		Aquilaria		Pinellia		Rhodiola		Cornus		Trachelospermum
	Gentiana		Nardostachys		Arisaema		Apis		Rubus		Speranskia
	Scutellaria		Santalum		Typhonium		Glycyrrhiza		Nelumbo		Stephania
	Coptis		Allium		Hippophae		Astragalus	15	**Wind-d, Dispelling**		Euonymus
	Lonicera	6	**Digestant**		Gleditsia		Pseudostellaria		Angelica		Corchorus
	Smilax		Crataegus		Fritillaria		Epimedium		Clematis		Campsis
	Sargentodoxa		Raphanus		Sargassum		Morinda		Piper		Cynanchum
	Paris		Hordeum		Bambusa		Gynostemma		Aconitum		Ipomoea
	Selenarctos	7	**Hemostatic**		Platycodon		Eucommia		Lycopodium		Rhinoceros
	Forsythia		Sophora		Ginkgo		Alpinia		Liquidambar		Anemone
	Lobelia		Imperata		Morus		Homo		Gentiana		Hypericum
	Taraxacum		Platycladus		Lepidium		Cuscuta		Morus		Toxicodendron
	Paeoniae		Panax	10	**Tranquillizing**		Cordyceps		Siegesbeckia		Castanea
	Rehmannia		Rubia		Polygonum		Cynomorium		Acanthopanax		Setaria
	Scrophularia		Typha		Ziziphus		Astragalus		Taxillus		Aralia
	Bubalus		Dalbergia		Platycladus		Dipsacus	16	**Dampness-R**		Crocus
	Artemisia		Bletilla		Polygala		Cistanche		Nelumbo		

Supplementary Table 3. Information of candidate anti-stroke compounds and their structural identify existing drugs. This table, all the compounds with TC value of 1 to drugs and their corresponding drugs information are shown, the information includes the ID, name, targets (plants). The drug information was found in DrugBank database, and the compound information are download from TCM database.

	Drug ID	Drug Name	Drug Target	Compound ID	Compound Name	Compound Target	Plant Name
1	DB00378	Dydrogesterone	Progesterone receptor	16186	Androst-4,6-diene-3,17-dione	COX-1	Moschus moschiferus
2	DB09124	Medrogestone	---				
3	DB00548	Azelaic Acid	Thioredoxin reductase, Tyrosinase, DNA polymerase I	26093	Aristolactam AIIIa	P2Y12	Aristolochia mollissima, Bombyx mori
4	DB00116	Tetrahydrofolic acid	Aminomethyltransferase	31953	2-Hydroxy-3-hydroxymethyl anthraquinone	COX-1	Morinda officinalis
5	DB00783	Estradiol	Estrogen receptor Nuclear receptor	18638, 18639	alpha-Estradiol, beta-Estradiol	P2Y12	Physostigma venenosum, Moschus berezovskii
6	DB00858	Drostanolone	Androgen receptor	26620	Blespirol	FXα, PARP1	Bletilla striata

7	DB01199	Tubocurarine	Acetylcholinesterase, 5-hydroxytryptamine receptor	19475,	Hayatidine	PARP1	Cissampelos pareira,
8	DB01336	Metocurine	Neuronal acetylcholine receptor, Muscarinic acetylcholine receptor	19476	Hayatine	PSD-95	
9	DB01586	Ursodeoxycholic acid	Aldo-keto reductase family	24295	Trihydroxybufosterocholanic acid	AChE	Coptis chinensis
10	DB02659	Cholic Acid	Ferrochelatase, Alcohol dehydrogenase, Estrogen-related receptor, Gastrotropin, Cytochrome c oxidase, Phospholipase, Liver carboxylesterase, Ferrochelatase, Choloylglycine hydrolase				
11	DB06777	Chenodeoxycholic acid	Bile acid receptor				
12	DB01744	Camphor	Camphor 5-monooxygenase	28170	Delajacine	SOD1	Delphinium giraldii
				16185	5 beta-Androstan-3, 17-dione	COX-1	Moschus moschiferus
13	DB08995	Diosmin	---	18311, 28958	Diosmin,	P53	Sophora vicllfolia, Taraxacum officinale, Salvia tomentosa
14	DB00696	Ergotamine	5-hydroxytryptamine receptor dopamine receptor adrenergic receptor noradrenaline transporter	18582	Ergotamine	PSD-95, PPARγ, PDE3,PARP-1, PDE5A, P53,ACE2, PAI-1, AChE, NOS3, FXαI	Claviceps purpurea
				18583	Ergotaminine		
15	DB00825	Menthol	Transient receptor potential cation channel subfamily, Kappa-type opioid receptor	18888	Friedelanol	PARP-1	Conyza blinii, Bischofia javanica
				18887	Friedelan-3α-ol	PSD-95	SUO LA MU

				32328	Hydroxyphaseollin	P53	Glycine max
**16**	DB01016	Glyburide	ATP-binding cassette, ATP-sensitive /G protein-activated inward rectifier potassium channel, Bile salt export pump, Cystic fibrosis transmembrane conductance regulator, Carnitine O-palmitoyltransferase, Mitochondrial ATP-sensitive potassium channel	30559	Genameside D	P2Y12<br>PAI-1<br>PPARγ	Jin ni pi

Supplementary Table 4. The 24 compounds passed ADMET filter. This table contains the information about ID, 2D structure, targets and corresponding plants of 24 compounds after ADMET filter. Aqueous solubility (**Sol.**): 2~4-Yes.but low, good, optimal; blood brain barrier penetration (**BBB**): 0~2-very high penetrant, high, low; human intestinal absorption (**Abs.**): 0~1-good, moderate; hepatotoxicity (**Hep.**): FALSE; plasma protein binding (**PPB**): FALSE.

Name (ID)	Structure	Sol.	BBB	Abs.	Hep.	PPB	Target
Sinapic acid-4-O-beta-glucuronide (6966)	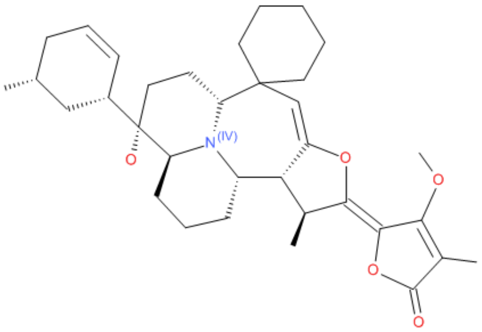	2	2	0	FALSE	FALSE	SOD1
1-O-beta-D-glucopyranosyl-(2S,3R,4E,8Z)-2-[(2R-hydroxyoctadecanoyl)amido]-4,8-octadecadiene- 1,3-diol (7026)	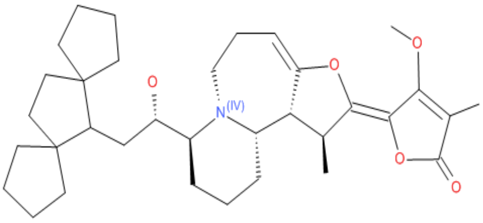	2	1	0	FALSE	FALSE	FXα

7040	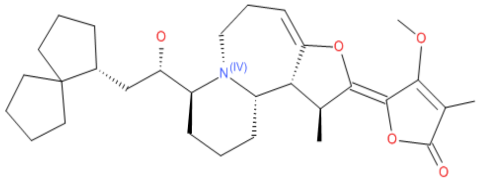	2	2	0	FALSE	FALSE	FXα
Acankoreoside D (7047)	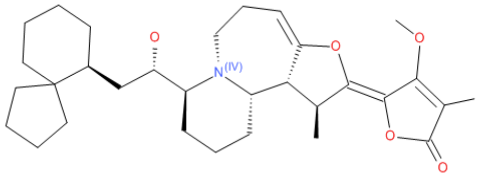	2	2	0	FALSE	FALSE	FXα
Dianthins,d (8619)	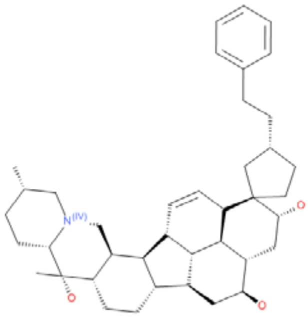	2	1	0	FALSE	FALSE	ACE2, PDE3, PDE5A ,PPARγ ,PSD-95, P53, PARP1
(S)-Suspensaside,Methyl,Ether (8625)	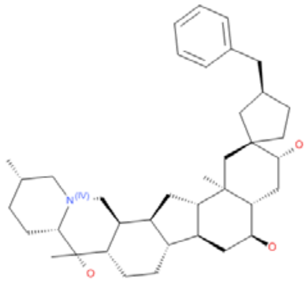	2	1	0	FALSE	FALSE	PDE3, PSD-95
Mimengoside,d (8773)	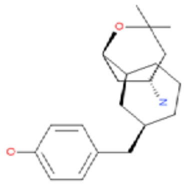	2	1	0	FALSE	FALSE	PAI-1
Shancilin (9438)	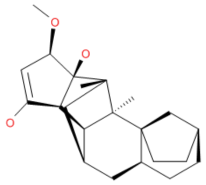	2	2	0	FALSE	FALSE	PPARγ, P2Y12
Tonkinochromane,c (9908)	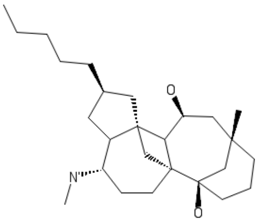	2	1	0	FALSE	FALSE	PDE3, PPARγ, P53, NOS3, AChE

(-)-Aptosimon (10418)	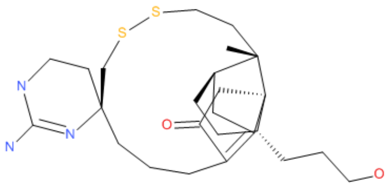	2	2	0	FALSE	FALSE	P2Y12
4,5-di-o-caffeoyl ,quinic,acid (10639)	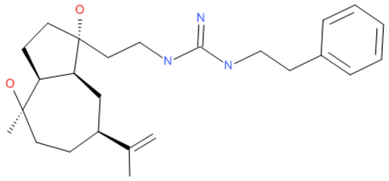	3	2	0	FALSE	FALSE	FII
Cordylagenin (17367)	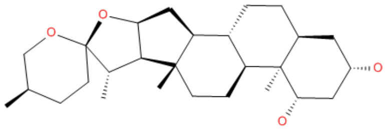	2	1	0	FALSE	FALSE	ACE2
Dihydroverticillatine(18121)	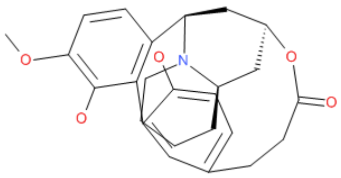	2	2	0	FALSE	FALSE	FII
Epirockogenin (18527)	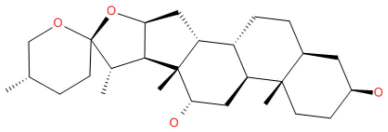	2	1	0	FALSE	FALSE	SOD1
12-Epirockogenin (18528)	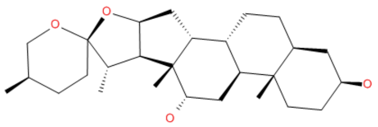	2	1	0	FALSE	FALSE	SOD1
Lythrancepine II (20799)	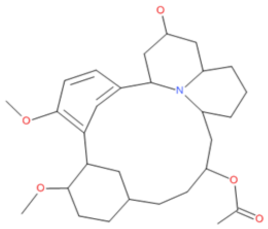	2	2	0	FALSE	FALSE	PARP1
Lythrancine II (20802)	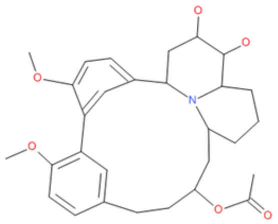	2	2	0	FALSE	FALSE	PARP1

Manogenin (20937)	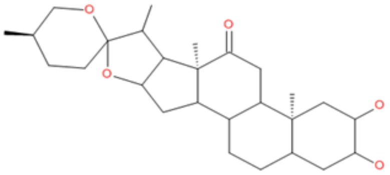	2	1	0	FALSE	FALSE	PARP1
Panaxatriol (22355)	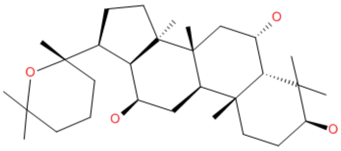	2	1	0	FALSE	FALSE	SOD1
Rhodeasapogenin (23071)	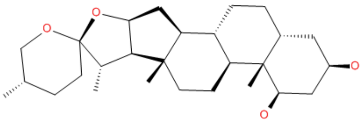	2	1	0	FALSE	FALSE	PARP1
Rockogenin (23116)	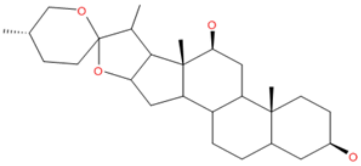	2	2	0	FALSE	FALSE	ACE2, PARP1
Tokorgenin (24194)	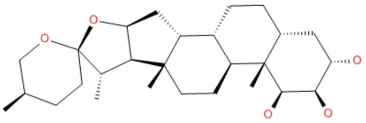	2	1	0	FALSE	FALSE	ACE2
Torvogenin (24216)	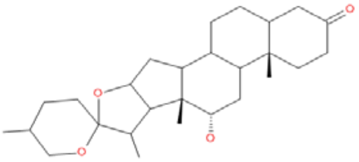	2	2	0	FALSE	FALSE	SOD1
Yomogin (24825)	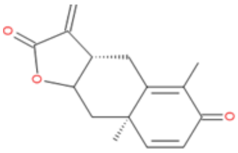	2	1	0	FALSE	FALSE	COX-1
Yonogenin (24826)	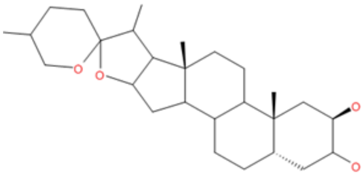	4	2	0	FALSE	FALSE	SOD1
Blestriarene A (26624)	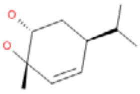	3	2	0	FALSE	FALSE	ACE2, PARP1
Blestrin D (26629)	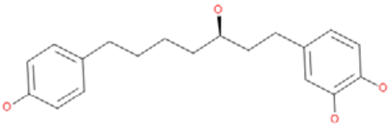	2	1	0	FALSE	FALSE	PDE3, PDE5A, AChE

Conyzasaponin A (27575)	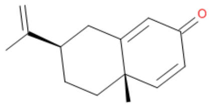	4	2	0	FALSE	FALSE	COX-1
Dibothrioclinin II (28468)	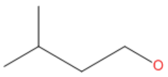	3	2	0	FALSE	FALSE	PSD-95, FII, PARP1
Glabroisoflavanone A (30713)	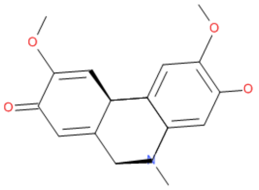	3	2	0	FALSE	FALSE	FII
Hupehenidine (31515)	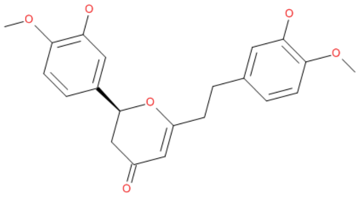	2	2	0	FALSE	FALSE	P2Y12
15α-Hydroxysoladulcidine (32421)	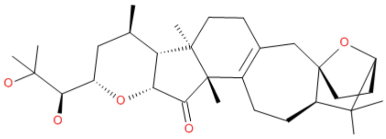	2	1	0	FALSE	FALSE	FXα
Ivangustin (33198)	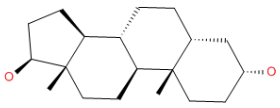	2	2	0	FALSE	FALSE	COX-1
Kaempferol-3-O-β-D-glucopyranosyl(1→2)-β-D-6-acetylglucopyranoside (33377)	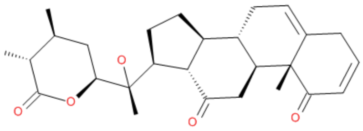	2	2	0	FALSE	FALSE	PPARγ, AChE
Prosapogenin CP5 (37343)	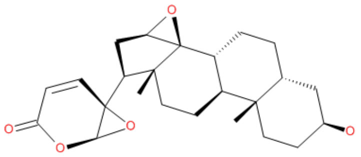	2	1	0	FALSE	FALSE	AChE

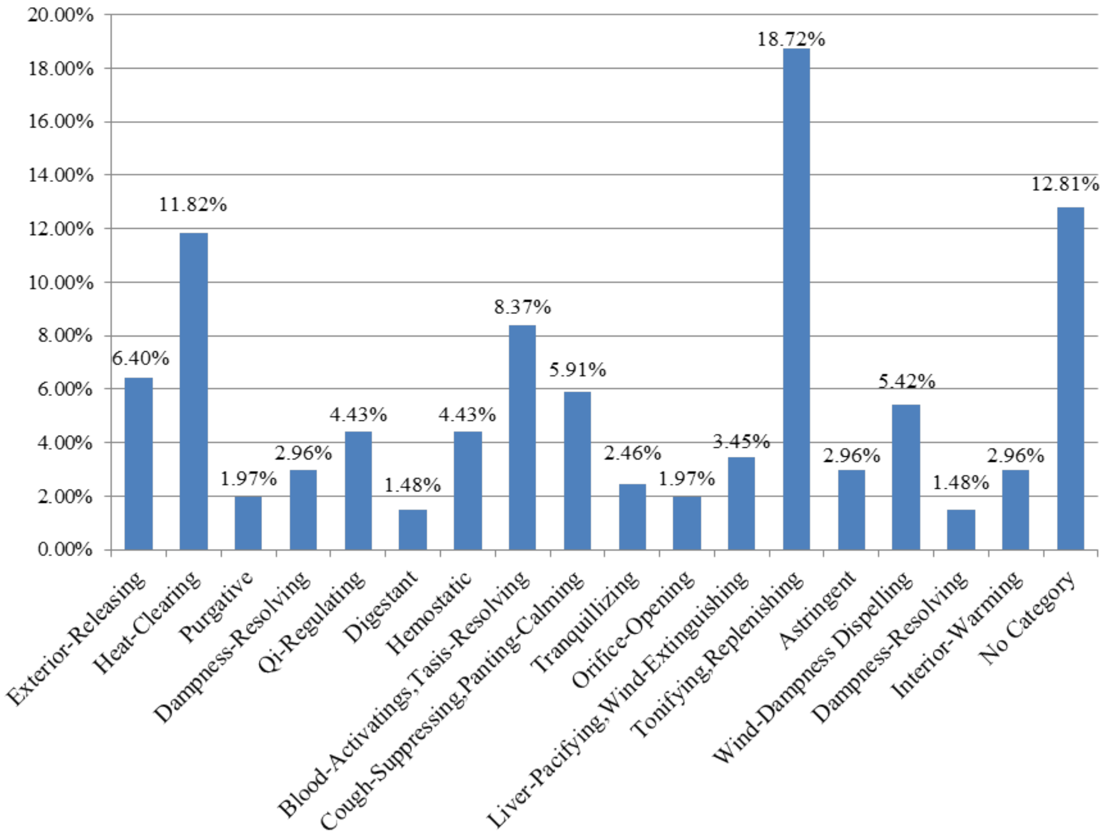

Supplementary Figure 1. The proportion of clinical anti-stroke plants in different properties. 192 anti-stroke plants are covered in 18 types, one of which is not defined.

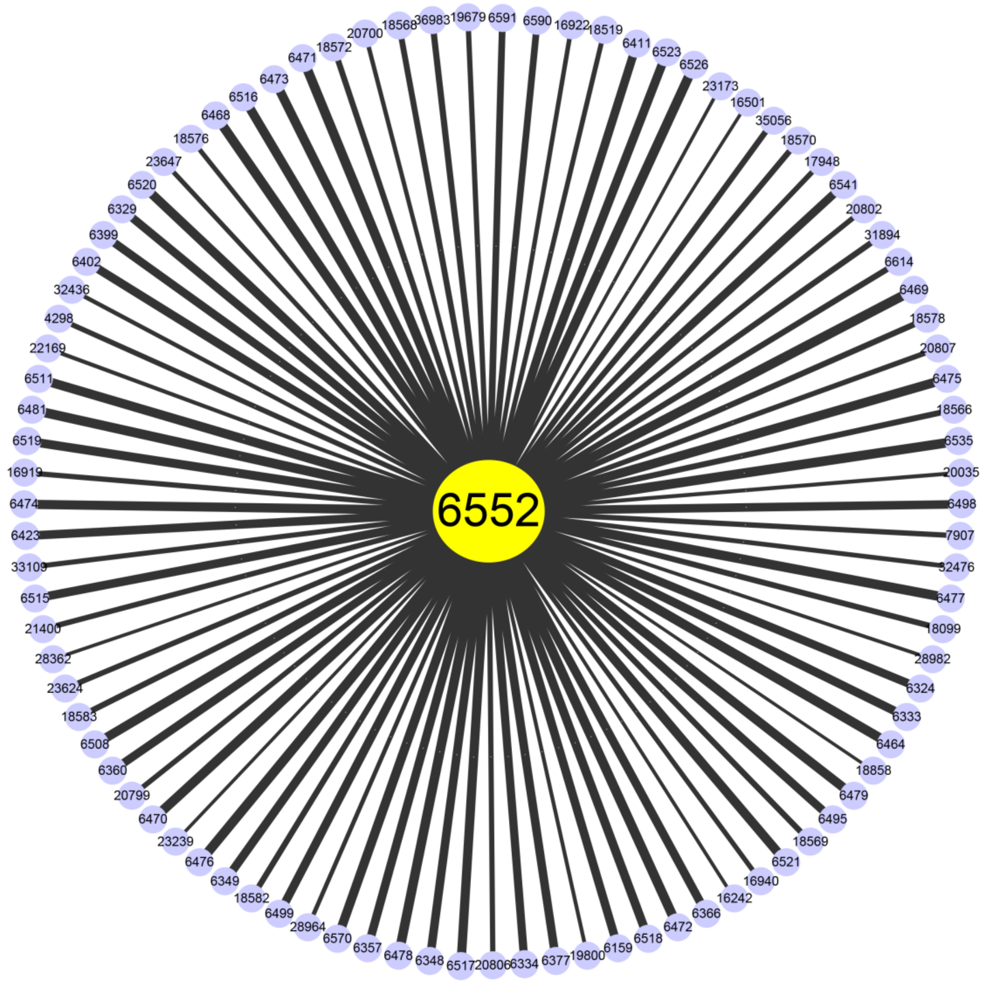

Supplementary Figure 2. One of the example display about the cluster condition of top 1% compounds. This picture show the relationship between cluster member (colored by purple) and cluster center molecular (colored by yellow) compound 6552 of cluster9 in figure 5. The thickness of lines represents the cluster molecular distance value which ranges from 0.34 (compound 18858) to 1 (compounds 6478, 6476, 6470, 6481, 6468, 6471, 6526, 6469, 6535, 6477, 6464, 6472).

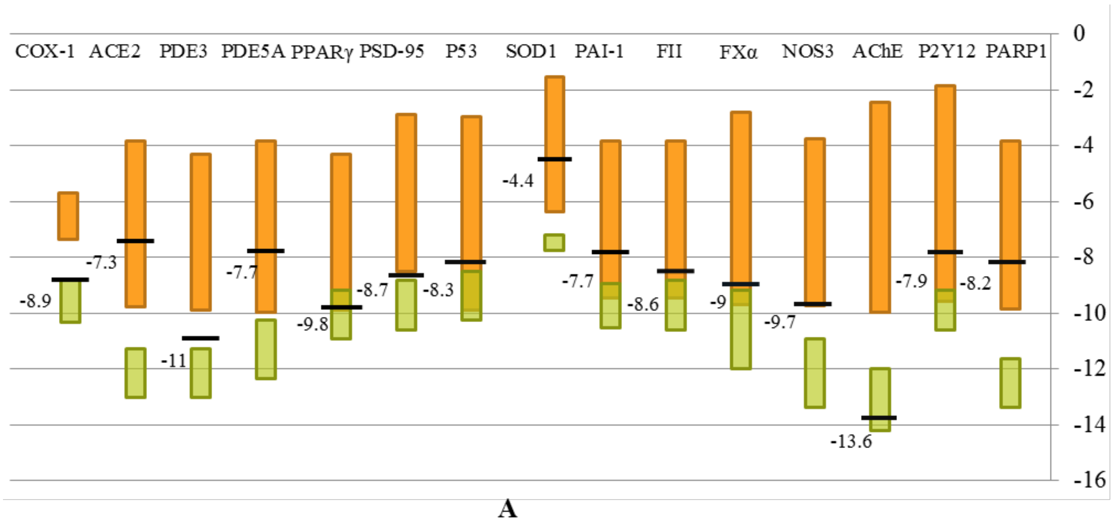

A

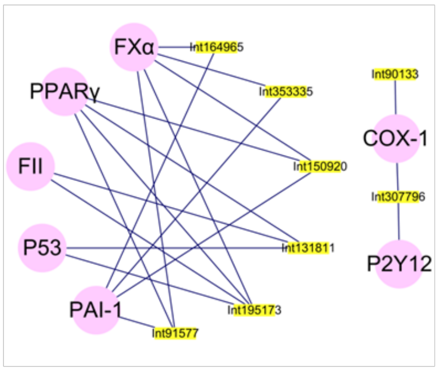

B

Supplementary Figure 3. The docking results of anti-stroke targets with Integrity drugs and top 1% compounds. A: Green and orange rectangles are respectively the docking value range of top 1% compounds and Integrity drugs. The black line means the docking energy of ligand which embedded in the crystal structure for each target. **B:** The interaction between Integrity drugs and anti-stroke targets. Drugs and targets are respectively colored by yellow and pink.
